# Toll-Like Receptors in Ischaemia and Its Potential Role in the Pathophysiology of Muscle Damage in Critical Limb Ischaemia

**DOI:** 10.1155/2012/121237

**Published:** 2012-02-07

**Authors:** Hemanshu Patel, Sidney G. Shaw, Xu Shi-Wen, David Abraham, Daryll M. Baker, Janice C. S. Tsui

**Affiliations:** ^1^Division of Surgery & Interventional Science, University College London, Royal Free Campus, London NW3 2QG, UK; ^2^Department of Clinical Experimental Research, University of Bern, 3010 Bern, Switzerland; ^3^Centre for Rheumatology and Connective Tissue Disease, University College London, Royal Free Campus, London, NW3 2QG, UK

## Abstract

Toll-like receptors (TLRs) are key receptors of the innate immune system which are expressed on immune and nonimmune cells. They are activated by both pathogen-associated molecular patterns and endogenous ligands. Activation of TLRs culminates in the release of proinflammatory cytokines, chemokines, and apoptosis. Ischaemia and ischaemia/reperfusion (I/R) injury are associated with significant inflammation and tissue damage. There is emerging evidence to suggest that TLRs are involved in mediating ischaemia-induced damage in several organs. Critical limb ischaemia (CLI) is the most severe form of peripheral arterial disease (PAD) and is associated with skeletal muscle damage and tissue loss; however its pathophysiology is poorly understood. This paper will underline the evidence implicating TLRs in the pathophysiology of cerebral, renal, hepatic, myocardial, and skeletal muscle ischaemia and I/R injury and discuss preliminary data that alludes to the potential role of TLRs in the pathophysiology of skeletal muscle damage in CLI.

## 1. Introduction

Critical limb ischaemia (CLI) is the most severe form of peripheral arterial disease (PAD). Whilst PAD describes stenotic or aneurysmal disease in any arterial bed except the coronary arteries, CLI generally describes advanced atherosclerosis in the lower limb arteries leading to a reduced blood supply to the tissues of the lower limb resulting in rest pain and/or tissue loss. PAD affects 27 million people in Western Europe and North America, and approximately 1-2% of these patients will develop CLI [1]. Further, CLI is associated with significant morbidity and mortality: a large observational multicentre cohort study of CLI patients observed a 6-month amputation rate of 12% and 1-year mortality rate of 19.1% [2]. However despite the importance of this condition, management of CLI patients continues to be challenging with limited treatment modalities available. Surgical or endovascular intervention remains the mainstay of therapy by improving blood flow. However, even successful revascularisation is not associated with an improvement in the functional ability of patients with CLI [3], and most patients have persistent or recurring symptoms requiring further treatment [4]. In addition, a significant number of patients with CLI are not suitable for revascularisation and treatment is limited to pharmacological agents such as iloprost where outcomes have been unsuccessful or inconsistent [5] or amputation. Whilst work has been carried out on the aetiology of PAD the downstream effects of reduced blood flow to the main organ, that is, skeletal muscle are still poorly understood. A better understanding of the pathophysiological processes occurring within the skeletal muscle in CLI may enable us to identify potential therapeutic targets. Recent studies on ischaemia and ischaemia/reperfusion (I/R) injury of various organs systems have identified the involvement of toll-like receptors (TLRs) in the pathogenesis of hypoxic/ischaemic injury [6]. We aim to review this evidence for the role of TLRs in ischaemia and ischaemia/reperfusion injury as well as discuss the potential implication of TLRs in the pathophysiology of skeletal muscle in CLI.

## 2. Toll-Like Receptors

Toll-like receptors are key receptors of the innate immune system as they recognise and respond to components of invading microorganisms termed pathogen-associated molecular patterns (PAMPs). PAMPs consist of lipids, lipopeptides, proteins, and nucleic acids [7], and upon binding to TLRs they lead to the activation of the TLR signalling pathway. This culminates in the release of various cytokines, chemokines, and interferons that has implications for both the innate and adaptive immune systems [8]. TLRs are type 1 membrane glycoproteins that consist of a ligand-binding external domain comprised of 19–25 leucine rich repeat (LRR) motifs and a cytoplasmic signalling domain that is termed the toll/interleukin 1 (TIR) domain. So far 13 TLRs have been identified in mammals of which eleven functional TLRs (TLR 1–11) have been discovered in humans. These can be subdivided by their subcellular localisation. TLR 1, 2, 4, 5, 6, and 10 are expressed on the cell surface whereas TLR 3, 7, 8, and 9 are located in the intracellular compartments, typically in the endosomes and the endoplasmic reticulum [9]. Toll-like receptors are expressed on both immune and nonimmune cells such as macrophages [10], neutrophils [10], B cells [11] as well as epithelial cells [12], myocytes [13], and skeletal muscle [14].

## 3. Toll-Like Receptor Ligands and Antagonists

In addition to PAMPS, TLRs are stimulated by host-derived molecules such as high mobility group box 1 protein (HMGB-1) [15]. TLRs achieve ligand specificity by receptor dimerization: almost all the TLRs form homodimers except TLR 1, 2, and 6 whilst TLR 2 can heterodimerise with either TLR 1 or 6 depending upon the ligand that is presented [16]. The exogenous TLR ligands can be subdivided into 3 groups (Table 1). The first group consists of lipids which are recognised by TLR 1, 2, 4, and 6, the second group are proteins which bind to TLR 5 and 11, and the third group consists of nucleic acids which activate the intracellular TLRs such as TLR 3, 7, 8, and 9. Recent studies have identified numerous host-derived ligands of TLRs that are released under certain physiological and pathophysiological conditions. These are also summarised in Table 1, however particularly interesting endogenous ligands are heat shock proteins (HSP) [17], hyaluronic acid [18], and HMGB-1. These ligands are secreted in states of shock or tissue injury and are therefore involved in activating TLRs in certain pathological conditions such as hypoxia and ischaemia.

The emerging importance of TLR activation in the pathogenesis of numerous conditions has led to the development of a number of synthetic TLR antagonists. So far these antagonists are structural analogues of agonists which prevent the stimulating ligand binding to the receptor [19]. Disease modulation by TLR antagonism is not only being studied in animal models but a number of clinical trials in humans are underway for the treatment of septic shock and autoimmune disorders. Nonetheless there are very limited TLR antagonists available at present, and a better understanding of TLR ligands will aid in the development of a broader spectrum of antagonists. In this respect, various groups are using screening techniques such as systemic evolution of ligands by exponential enrichment (SELEX) and high-throughput screening (HTS) to identify and develop oligonucleotides and synthetic phospholipid compounds with the potential to inhibit TLR signalling.

## 4. TLR Signalling

Stimulation of TLRs upon ligand recognition leads to the activation of its downstream signalling cascade which culminates in the activation of the transcription factors, nuclear factor-*κ*B (NF-*κ*B), and activator protein 1 (AP-1). This results in the release of various proinflammatory cytokines such as IL-6, IL-1, and TNF-*α*. The initiation of this signalling cascade depends upon the binding of the TIR domain of the intracellular portion of TLRs to the TIR domain-containing cytosolic adapter proteins. The four main adapter proteins are myeloid differentiation primary response protein 88 (MyD88), TIR domain-containing adapter protein (TIRAP/Mal), TIR domain-containing adapter-inducing IFN*β* (TRIF, also known as TICAM1), and TRIF-related adapter molecule (TRAM, also known as TICAM2) [20]. Also important for TLR signalling transduction is the interleukin-1 receptor-associated kinase (IRAK) family of which four members have so far be identified: IRAK1, IRAK2, IRAK3, and IRAK-M. Two separate signalling pathways exist for the signalling transduction of TLRs: these are termed the MyD88-dependent pathway of which MyD88 plays a central role and the MyD88-independent pathway which uses TRIF instead. All TLRs utilise the MyD88-dependent pathway except TLR3; interestingly TLR4 is capable of using either signalling pathway.

**Figure 1:**
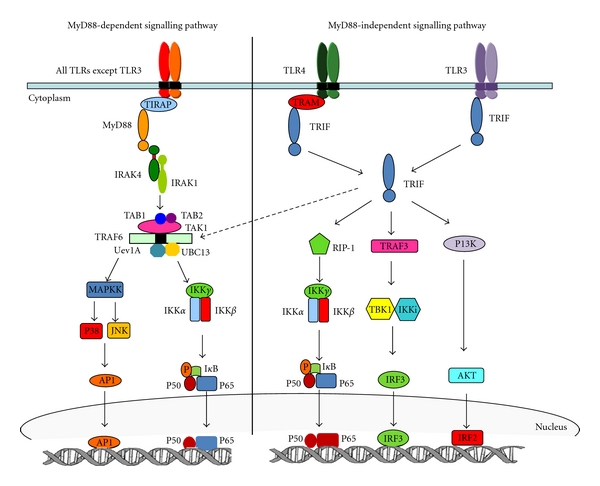
TLR signalling pathway. MyD88-dependent signalling pathway is used by all TLRs except TLR 3. Signalling through the MyD88-dependent pathway leads to the activation of MAPKK and IKK complex resulting in activation and nuclear translocation of AP-1 and NF-*κ*B, respectively. TLR 4 is capable of signalling through the MyD88-independent pathway as well; however this is the sole signalling mechanism for TLR 3. TRIF is the main adaptor protein in the MyD88-independent pathway and can associate with TRAF6 to activate AP-1 and NF-*κ*B. Alternatively it also activates NF-*κ*B by interacting with RIP-1. TRIF can further interact with TRAF3 and the phosphatidylinositol 3-kinase (PI3K)-AKT pathway resulting in the nuclear translocation of IRF3 and IRF2, respectively.

In the MyD88-dependent pathway (Figure 1), ligand binding leads to the association of MyD88 to the TIR domain of the receptor. This leads to the phosphorylation of IRAK4 which in turn phosphorylates IRAK1. IRAK1 binds to and activates tumour necrosis factor receptor-associated factor 6 (TRAF6). The IRAK1/TRAF6 complex dissociates from the intracellular receptor complex and forms a complex with the E2 ligases Ubc13 and Uev1A which have been shown to catalyse the synthesis of a Lys 63-linked polyubiquitin chain of TRAF6. This complex further associates with a member of the MAP kinase kinase kinase family, transforming growth factor-*β*-activated kinase 1 (TAK1) and the TAK1-binding proteins, TAB1 and TAB2. This complex formation subsequently activates TAK1 which simultaneously activates the I*κ*B kinases (IKK) complex and members of the MAP kinase family such as extracellular signal-regulated kinase (ERK), c-Jun kinase (JNK), and p38. The IKK complex consists of two kinases, IKK*α* and IKK*β* and a regulatory subunit (NEMO/IKK*γ*) [21]. IKK-activated complex subsequently phosphorylates the inhibitory I*κ*B proteins which normally sequester NF-*κ*B in an inactive form in the cytoplasm. I*κ*B protein phosphorylation leads to their polyubiquitylation and subsequent degradation with the concomitant release and nuclear translocation of NF-*κ*B. The activation of members of the MAPK family such as JNK leads to the subsequent phosphorylation and activation of the transcription factor AP-1. Further, stimulation of the MyD88-dependent pathway in TLR 7, 8, and 9 can also lead to the induction of type 1 interferons(IFN) possibly through the activation of interferon regulatory factor 7 (IRF7) [22]. 

The MyD88-independent pathway which is the sole signalling transduction system of TLR 3 and is an alternative signalling pathway for TLR 4 also culminates in the activation of NF-*κ*B and AP-1 as well as IRF3 leading to the induction of type 1 interferons. In the MyD88-independent pathway TRIF is the main adaptor protein. In TLR 3 signalling TRIF binds directly to the cytoplasmic portion of the receptor; however in TLR 4 signalling the adaptor protein TRAM acts as a bridge between TRIF and the TIR-containing domain. TRIF can interact with TRAF6 or receptor-interacting protein-1 (RIP-1) leading to the activation of NF-*κ*B and AP-1. Further TRIF leads to the phosphorylation and the consequent activation of IRF 3 and 7 leading to the induction of type 1 IFNs. Two members of the IKK family IKKi (IKK*ε*) and TBK1 [TRAF family member-associated NF-*κ*B activator (TANK) binding kinase-1 or T2K or NAK] are however essential to this role [23, 24].

## 5. Ischaemia and Ischaemia/Reperfusion Injury

The pathophysiology of ischaemia and ischaemia/reperfusion- (I/R-) induced injury has been extensively studied and evaluated in various organ systems such as the kidneys, liver, cardiovascular, and central nervous systems (CNS) (Table 2). There is growing evidence that TLRs play an important role in the propagation of the tissue damage caused by ischaemia and I/R. However there is still a lack of significant progress in understanding the pathophysiology of skeletal muscle damage in CLI and the role TLRs play in this disease process. Following ischaemia or I/R a number of cellular and biochemical changes occur both locally and systemically. This include the recruitment of activated neutrophils [25] and lymphocytes [26], production of reactive oxygen species (ROS), release of cytokines [27] and chemokines [28], and activation of the complement system [29]. The generation of ROS, ATP deletion, and activation of enzymes such as phospholipases and proteases lead to cell necrosis. Apoptosis has also been shown to occur following ischaemia [30], and it is thought that mitochondrial dysfunction secondary to ROS generation plays a role in inducing the apoptotic mechanism. In addition, TLRs seem to play an important role in mediating some of the ischaemia-induced injury. Endogenous ligands of TLRs such as fibrinogen, heparin sulphate, hyaluronan, HSP60, HSP70, and HMGB-1 are released by injured and necrotic cells. The subsequent stimulation of TLRs by these ligands leads to the activation of transcription factors NF-*κ*B and AP-1 with a consequent release of proinflammatory cytokines. Further TLRs have been implicated in directly causing apoptosis via a pathway involving Fas-associated death domain protein (FADD) and caspase 8 [31].

## 6. The Role of Toll-Like Receptors in the Pathophysiology of Cerebral Ischaemia and I/R Injury

There is emerging evidence that TLRs play a role in neuronal damage secondary to cerebral ischaemia. TLRs are thought to be expressed primarily by the glial cells (microglia, astrocytes, and oligodendrocytes) in the brain. Human microglia are found to express TLRs 1–8 at detectable levels and TLR 9 at low but detectable levels [32]. The expression of TLRs in human astrocytes, however, is more restricted with only TLR 3 mRNA detected at intermediate levels; mRNA for TLRs 1, 2, 4, 5, and 9 were detectable but were low, and TLRs 6, 7, 8, and 10 mRNA expression was found to be rare to undetectable [33]. Very little is known about the expression of TLRs in human oligodendrocytes, but Bsibsi et al. [32] have reported expression of TLRs 2 and 3 in these cells. TLRs 2 and 4 have also been found to be expressed in mouse cerebral cortical neurons [34]. When microglia and astrocytes are exposed to TLR ligands such as peptidoglycan, double-stranded RNA, lipopolysaccharide, and bacterial DNA there is a release of a wide range of proinflammatory cytokines (including TNF-*α*, IL-6, and IL-12), chemokines, and reactive oxygen species. This suggests that the TLRs are involved in protecting the CNS against microbial infections, although it is unclear whether this neuroinflammatory response is beneficial or detrimental. Growing evidence suggests that the TLRs expressed in the CNS also play an important role in tissue development, cellular migration and differentiation, and in limiting inflammation [66].

 Acute inflammation exacerbates brain damage in cerebral ischaemia, and activation of the innate immune system is an important component of this process. There is now strong evidence suggesting that TLRs within the CNS play an integral part in this inflammatory process. For example TLRs 2, 4, and 9 have been shown to be upregulated and activated in cerebral ischaemia models [34, 35]. Significantly a number of groups have reported reduced infarct size in TLR 2 and 4 knockout mice that have been exposed to cerebral ischaemia [34, 36–38]. Kilic et al. [36] performed intraluminal middle cerebral artery occlusion as a model of ischaemic stroke in adult male C3H/HEJ TLR 4 knockout mice. They showed that in the TLR4-deficient mice there was reduced ischaemic neuronal injury, and the mechanism of this neuroprotective effect was associated with deactivation of the MAP kinases ERK 1, ERK2, JNK1, JNK2, and P38 [36]. Further, Caso et al. [67] not only demonstrated reduced infarct size but also recovery of neurological deficit in TLR 4 knockout mice that had MCA occlusion suggesting actual clinical improvement as a result of abolishing the effects of TLR 4. Interestingly, studies have shown that LPS (TLR 4 ligand) pre-conditioning helps to protect against subsequent ischaemic damage; however the mechanism for this is unclear. Thus, there is now significant evidence that TLRs in particular TLR 2 and 4 play a role in ischaemia-induced cerebral damage but the exact mechanisms are yet to be elucidated. Apart from the increase in proinflammatory cytokines, activated glial TLRs also lead to the release of chemokines such as macrophage inflammatory protein-2 (MIP-2) and monocyte chemoattractant protein-1 (MCP-1) which attract peripheral immune cells into the brain parenchyma and may lead to further exacerbation of ischaemic damage. Using a model of glucose deprivation as a form of energy deprivation, Tang et al. [34] found that TLR2 and 4 are implicated in apoptotic neuronal cell death via activation of the JNK-AP-1 pathway. In addition they showed that the endogenous ligand of TLR 2 and 4, HSP70, is upregulated thus implicating HSP70 and other endogenous ligands of TLRs in the pathogenesis of cerebral ischaemia.

## 7. The Role of Toll-Like Receptors in the Pathophysiology of Liver Ischaemia and I/R Injury

Liver ischaemia and reperfusion can occur during a variety of situations particularly during surgical procedures such as liver transplant, vascular reconstruction, liver trauma, and resection of large hepatic tumours. During the initial ischaemic period a certain level of cellular damage has been shown to occur [68]; however this is further exacerbated following reperfusion [69]. The liver is primarily composed of parenchymal cells (hepatocytes) constituting 65% of the cells in the liver. The remaining part of the liver is composed of nonparenchymal cells such as kupffer cells, sinusoidal endothelial cells, biliary epithelial cells, hepatic stellate, and dendritic cells. Studies have shown that both the parenchymal and nonparenchymal cells express a large repertoire of TLRs [39].

Kupffer cells which are the resident macrophages of the liver play a central role in hepatic I/R injury. Following hepatic ischaemia kupffer cells are activated and lead to the release of both proinflammatory cytokines (TNF-*α*, IL-6, and IL-1) as well as anti-inflammatory cytokines (IL-10 and IL-13) and ROS [70]. Inflammatory cytokines both initiate and maintain the inflammatory response and, together with the activated complement system, result in the migration and adhesion of leukocytes and recruitment of neutrophils within the sinusoids [71]. Activated neutrophils further exacerbate liver damage induced by reperfusion, through the release of more ROS and proteases [72]. The resulting increase in ROS causes oxidative stress and cell death. Significantly, ROS especially hydrogen peroxide [73] activate NF-*κ*B which has been found to play a significant role in liver I/R injury [74]. Cell death in liver I/R has been shown to occur by both apoptosis [75] and necrosis [76]. Inflammatory mediators such as TNF-*α* released by kupffer cells and neutrophils have been shown to activate pro-apoptotic proteins such as caspase-3 and caspase-8 further highlighting the role of these cells in ischaemic liver damage [77].

Several groups have illustrated the importance of TLR signalling in hepatic I/R damage [40–42, 45]. In particular TLR 4 and 9 are specifically implicated in the pathological process. Tsung et al. [41] showed that chimeric mice lacking functional TLR 4 subjected to liver I/R were protected from tissue damage. Further they showed that this protective effect was associated with a reduction in the activation of JNK and NF-*κ*B as well as a decrease in the expression of the proinflammatory cytokines TNF-*α* and IL-6 and the adhesion molecule ICAM-1. Shen et al. [43] also showed that TLR 4 knockout mice were protected against hepatic I/R injury, and this was associated with a reduction in local and systemic TNF-*α* levels as well as reduced neutrophil infiltration.

Circulating levels of HSP72 have shown to be increased during hepatic I/R injury [44, 78]. HSP72 stimulated both TLR 2 and 4 in hepatocytes leading to the activation of NF-*κ*B and the subsequent production of macrophage inflammatory protein-2 (MIP-2) but not TNF-*α* or IL-6. MIP-2 promotes neutrophil infiltration during hepatic I/R injury, and blockade of MIP-2 has been shown to reduce hepatic I/R injury [79]. In TLR 2 and 4 knockout mice, MIP-2 production is reduced suggesting that both TLR 2 and 4 contribute to hepatic I/R injury [78]. However, despite an increase in TLR 2 mRNA levels after hepatic I/R injury [40] it has been found that TLR 4 but not TLR 2 is required in initiating the hepatic injury cascade as it was demonstrated that TLR 2 knockout mice and wild-type mice livers suffered comparable I/R injury [80]. Further, Zhai et al. [80] have suggested that TLR 4-induced hepatic damage in I/R is mediated through the MyD88-independent pathway rather than the MyD88-dependant pathway. They showed that IRF 3-deficient mice protected their livers from I/R injury in a similar fashion to TLR 4-deficient mice but in MyD88-deficient mice significant hepatic I/R injury still occurred. This finding however cannot account for the increase in the proinflammatory cytokines which occurs during liver ischaemia and may be explained by the work carried out by Bamboat et al. [42] demonstrating that TLR 9 may also be involved in hepatic I/R injury. They reported that following I/R TLR 9 knockout mice showed minimal hepatic damage associated with reduced proinflammatory cytokine levels (TNF-*α*, IL-6, and MCP-1) compared to wild-type mice which exhibited severe hepatocellular necrosis. TLR 9 blockade also reduced liver damage and cytokine levels in wild-type mice that were subjected to I/R. This suggests that both TLR 4 and 9 play an important role in hepatic damage secondary to ischaemia and I/R injury and that TLR 9 stimulation may mediate the increase in proinflammatory cytokines.

In addition to HSP72, other endogenous TLR ligands may be involved in hepatic I/R. Bamboat et al. [42] have shown that DNA from necrotic hepatocytes stimulate TLR 9 leading to cytokine release. HMGB-1 is a DNA-binding protein which is released by necrotic cells including hepatocytes. HMGB-1 levels increase during liver I/R, and inhibition of HMGB-1 with a neutralising antibody reduces liver damage after I/R [42, 45]. Thus, numerous TLR endogenous ligands have been implicated in hepatic I/R injury; however it remains to be seen how much each of them contributes to hepatic I/R injury.

## 8. The Role of Toll-Like Receptors in the Pathophysiology of Renal Ischaemia and I/R Injury

Renal ischaemia and I/R injury can occur during transplantation, partial nephrectomy, aortic cross-clamping, and following systemic hypotension and is a common cause of acute renal failure (ARF). The pathogenesis of ischaemia and I/R-induced renal injury is complex and incompletely understood. However, it is not surprising that the innate immune system plays a significant role in the injury process. Ischaemia leads to the depletion of cellular ATP, and this causes tubular epithelial cells to undergo necrosis or apoptosis [81]. In addition to the cytotoxic effects of hypoxia, I/R triggers numerous inflammatory events. The renal endothelial and epithelial cells release inflammatory cytokines (IL-1, IL-6 and TNF-*α*) [82] as well as chemokines and express adhesion molecules that activate lymphocytes. Further, the infiltrating leukocytes also generate cytokines and ROS that exacerbate cellular injury. Several studies have also highlighted the importance of activation of the complement system [83], neutrophils [84], B cells [85], and T cells [86] in the development of renal I/R injury.

TLR expression in the kidney has been studied by several groups and mRNA for almost all the TLRs have been detected in human kidneys [46–48]. However TLRs 1, 2, 3, 4, 5, and 7 were found to be more abundantly expressed than TLRs 6, 8, 9, and 10. TLR 2 and 4 are expressed in mouse renal cortex and medulla, specifically in the proximal and distal tubules as well as in the epithelium of Bowman's capsule [47]. Recent studies have implicated TLR 2 and 4 in the pathogenesis of renal ischaemia and I/R injury. It has been shown that TLR 2 and 4 mRNA is upregulated following I/R in the epithelial cells of the distal tubules, thin limb of the loops of Henle, and collecting ducts. This upregulation was shown to be mediated by IFN-*γ* and TNF-*α* [47]. It has been found that TLR 2 knockout mice are protected against renal I/R injury [50, 51]. Leemans et al. [51] used TLR 2 antisense oligonucleotide treatment to reduce TLR 2 protein in mice kidney and showed that this also protected the kidney against I/R injury by demonstrating reduced renal dysfunction. The detrimental effects of TLR 2 signalling in renal I/R injury were observed to be due to an increase in chemokine and cytokine (MIP-2, TNF-*α*, IL-6, IL-1*β*) production, granulocyte and macrophage infiltration, as well as tubular necrosis, and tubular epithelial cell apoptosis, which was mediated through nonhematopoietic cells of the kidney. Interestingly Shigeoka et al. [87] have suggested a fascinating concept that renal damage in I/R due to TLR 2 activation is mediated through both a MyD88-dependent pathway as well as a TLR 2-dependent/MyD88-independent pathway.

TLR 4 has also been shown to play a significant role in renal I/R injury. Wu et al. [49] have demonstrated increased TLR 4 expression following kidney ischaemia. As demonstrated in TLR 2 knockout mice, it has also been shown that TLR 4 knockout mice are protected against renal dysfunction following I/R injury [49, 50, 52]. Further TLR 4 knockout mice subjected to I/R showed reduced tubular damage, neutrophil and macrophage accumulation, and inflammatory cytokine and chemokine production*. In vitro*, wild-type kidney tubular epithelial cells (TECs) that were subjected to ischaemia produced inflammatory cytokines and chemokines and underwent apoptosis. These effects were attenuated in TLR4^−/−^ and MyD88^−/−^ TECs [49]. These results provide significant evidence for the role of TLR 2 and 4 in the pathogenesis of ischaemia and I/R-mediated renal damage. A number of studies have demonstrated an upregulation of several TLR 2 and 4 endogenous ligands such as HMGB-1, hyaluronan and biglycan in the kidney during I/R injury [49, 51, 53] which may be involved in the activation of these receptors and subsequent inflammatory response and tissue damage in renal I/R.

## 9. The Role of Toll-Like Receptors in the Pathophysiology of Myocardial Ischaemia and I/R Injury

Myocardial ischaemia is most commonly due to occlusion of a major coronary artery. Coronary artery occlusion and the consequent reduction in blood flow usually occur due to fissuring or erosion of an atherosclerotic plaque with subsequent formation of thrombus. The pathogenesis of ischaemia-induced myocardial damage has been intensively studied, and a number of biochemical and cellular mechanisms have been discovered. Oxygen deficiency induces metabolic changes such as decreased ATP and pH as well as lactate accumulation. The altered biochemical status leads to impaired membrane transport resulting in an imbalance in intracellular electrolytes and propagates various other pathological metabolic changes resulting in cardiomyocyte death through necrosis and apoptosis. Irreversible damage occurs after approximately 30 minutes of coronary artery occlusion. Reperfusion exacerbates myocardial damage which is predominantly the effect of oxygen radicals, calcium loading, and neutrophil activation. Oxygen radicals cause further membrane damage, and neutrophils release inflammatory mediators and contribute to microvascular obstruction [88–90]. Inflammatory cytokines such as IL-6, TNF-*α*, IL-1*β*, and IL-8 have been shown to be upregulated during periods of myocardial ischaemia [91–93].

Most of the TLRs are expressed within the cardiovascular system. In particular, TLR 2, 3, 4, and 6 are expressed in rat cardiomyocytes [94] whilst both healthy and atherosclerotic arteries express TLRs 1–9 [55]. TLR 2 and 4 have been strongly implicated in myocardial damage following ischaemia and reperfusion. Murine TLR 4 expression has been shown to be increased after myocardial infarction [56], and various studies have shown that TLR 4-deficient mice have reduced myocardial infarct size when compared with control mice [57, 58, 95]. Studies have shown that the reduction in myocardial infarct size in TLR 4-deficient mice is attributed to a reduction in neutrophil infiltration and reduced JNK, NF-*κ*B, and AP-1 activation with a subsequent decrease in the levels of inflammatory cytokines (IL-1*β* and Il-6) and monocyte chemotactic factor-1 [57, 60, 95]. The P13K/AKT pathway has also been reported to play a role in protecting against myocardial I/R injury in TLR 4-deficient mice [96]. Mice pretreated with the TLR 4 antagonist eritoran prior to transient occlusion of the left anterior descending artery were shown to develop significantly smaller infarcts compared to mice treated with vehicle alone. Further, eritoran pretreatment resulted in reduced JNK phosphorylation, NF-*κ*B nuclear translocation, and proinflammatory cytokine expression [59]. 

There is also emerging evidence for the role of TLR 2 in myocardial I/R Injury. TLR 2 knockout mice subjected to myocardial infarction have been shown to have a higher survival rate than wild-type mice associated with a smaller infarct size, reduced ROS production, and leukocyte infiltration [97, 98]. Further, both bone marrow chimeric mice developed by transplanting TLR 2 knockout bone marrow to WT mice or WT bone marrow to TLR 2 knockout mice submitted to I/R 5 weeks after transplant displayed similar protection as TLR2^−/−^ mice against I/R-induced endothelial dysfunction, suggesting a role for TLR 2 expressed on both non-bone marrow cells such as endothelial cells and/or cardiomyocytes and cells of bone marrow origin such as neutrophils [98]. TLR 2 deficiency also abolished increased IL-1*β* expression but did not affect TNF-*α* or IL-6 expression. These studies provide some insight into the role of TLR 2 and 4 in myocardial I/R injury and may reveal potential therapeutic targets. 

HMBG-1 may again act as an endogenous TLR ligand in myocardial ischaemia. Andrassy et al. [99] demonstrated elevated levels of HMGB-1 following hypoxia in cardiomyocytes *in vitro *and in ischaemic injury of the heart *in vivo*. They also reported that treatment with recombinant HMGB-1 worsens I/R injury, whereas treatment with the HMGB-1 antagonist HMGB-1 box A reduced infarct size and markers of tissue damage. In addition their data suggested that HMGB-1-mediated myocardial damage involved JNK, ERK 1 and 2, and NF-*κ*B activation. Several other known HMGB-1-inhibiting agents (ethyl pyruvate, green tea, and adrenomedullin) have also been shown to preserve cardiac function following a myocardial ischaemic insult [54, 100, 101]. Further studies may reveal the involvement of other potential endogenous ligands in the setting of myocardial ischaemia.

## 10. The Potential Role of Toll-Like Receptors in the Pathophysiology of Critical Limb Ischaemia

CLI is a severe form of PAD that is predominantly caused by atherosclerosis in the peripheral arterial system. Whilst the detailed pathophysiology of CLI is not well understood, there is a basic understanding of the pathogenesis of skeletal muscle damage in peripheral arterial disease (PAD) and in I/R injury. A large proportion of mass in the limb is comprised of skeletal muscle, and therefore it is affected significantly by ischaemia-induced tissue damage. Studies have shown that irreversible muscle damage begins to occur after 3 hours of ischaemia and is nearly complete after 6 hours [102]. Pipinos et al. [103] have proposed a potential pathway for the pathogenesis of PAD-induced manifestations such as skeletal muscle damage and tissue loss, where inflammation, neutrophil activation and degranulation, and mitochondrial dysfunction with excessive production of ROS occur when blood supply is critically compromised. The consequences of these pathological processes include reduced energy production and damage to muscle as well nerves, skin, and subcutaneous tissue. Indeed, Hayes et al. [104] found that after a sustained period of ischaemia, diminishing ATP levels correlated closely with worsening muscle necrosis. Further, gastrocnemius muscle biopsies obtained from patients with PAD contain a higher number of apoptotic cells compared to controls as shown by Terminal Deoxynucleotidyl Transferase Biotin-dUTP Nick-end Labelling- (TUNEL-) staining and increased Caspase-3 activity [105]. Raised circulating levels of proinflammatory cytokines (TNF-*α*; 14.48 ng/ml versus 9.32 ng/ml and IL-6; 11.81 ng/ml versus 7.30 ng/ml), chemokines (vascular cell adhesion molecule-1 (VCAM-1); 485.09 ng/ml versus 464.35 ng/ml, and inter-cellular adhesion molecule-1 (ICAM-1); 316.7 ng/ml versus 207.65 ng/ml) are found in the plasma of patients with PAD [106]. Significantly, TNF-*α* and IL-6 have been shown to induce muscle proteolysis, and this in turn has been associated with reduced muscle mass and strength [62, 107, 108]. In addition TNF-*α* has also been reported to induce apoptosis in skeletal myoblasts [63]. It is therefore possible that the elevated levels of cytokines play a role in skeletal muscle damage in CLI.

**Figure 2:**
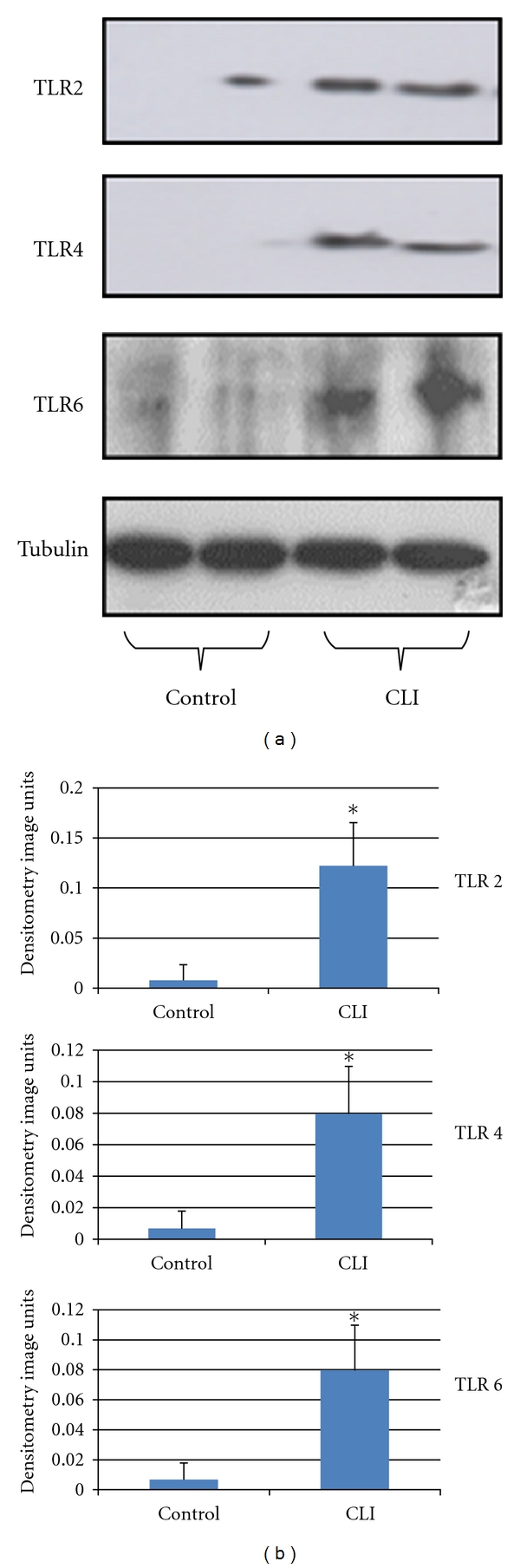
Representative western blots showing (a) increased TLR 2, TLR 4, and TLR 6 protein expression in gastrocnemius muscle biopsies obtained from patients with CLI compared to controls. (b) Densitometric quantification of TLR 2, 4, and 6 levels in CLI muscle. *P < 0.05 compared to control. The human experiments were conducted in accordance with the Declaration of Helsinki (1964), and informed consent was obtained from patients with prior approval from the local ethics committee.

TLRs 1–9 have been shown to be expressed in skeletal muscle [61, 64, 109]. Further, there is evidence to suggest that some of the TLRs are functional as lipopolysaccharide (LPS) and a synthetic tripalmitoylated cysteine-, serine-, and lysine-containing peptide (Pam), TLR 4, and TLR 2 ligands, respectively, induce TNF-*α*, IL-6, and IL-1*β* mRNA expression in gastrocnemius muscle and C2C12 myotubes [64, 109]. Further IL-6 induction has been shown to be mediated via activation of NF-*κ*B [62]. Warren et al. [61] demonstrated that TLRs 2, 4, 6, 8, and 9 are upregulated following freeze-induced skeletal muscle damage. TLR endogenous ligands such as HMGB-1 and HSPs have been shown to be expressed in skeletal muscle. HMGB-1 has been reported to induce skeletal muscle damage in inflammatory myopathies, and the expression of HSPs is upregulated in skeletal muscle following hypoxia and ATP depletion [110, 111]. We have recently found upregulation of TLRs 2, 4, and 6 protein expression in muscle biopsies obtained from patients with CLI (Figure 2). This indicates that TLRs are likely to be involved in the pathophysiology of CLI, possibly by contributing to the tissue damage that occurs, and provides the rationale to further elucidate the role of TLRs in the pathogenesis of skeletal muscle damage in CLI.

## 11. Conclusion

It can be concluded that following ischaemia and I/R significant tissue damage occurs in a number of different organs. The intricate mechanisms involved may differ depending on the type and duration of insult. However it is clear that inflammation following immune cell infiltration and ROS generation play a significant role in mediating cytotoxicity. There is ample evidence that TLRs of both immune and nonimmune cell origin are upregulated and activated in ischaemia leading to the production of various proinflammatory cytokines and chemokines. Both the MyD88-dependent and MyD88-independent TLR signalling pathways may be involved in mediating the ischaemic tissue damage, and the dominant signalling cascade used may be organ specific. TLRs have also been implicated in apoptotic cell death which plays a large part in ischaemia-induced cell damage. Necrotic cell death has also been shown to occur following ischaemia, and this is important as numerous TLR endogenous ligands such as HSP60 and HMGB-1 have been shown to be released during this process. This phenomenon may explain why TLRs are activated in sterile inflammation during ischaemia and provide an opportunity to manipulate the pathophysiological process in order to reduce cell damage. 

**Figure 3:**
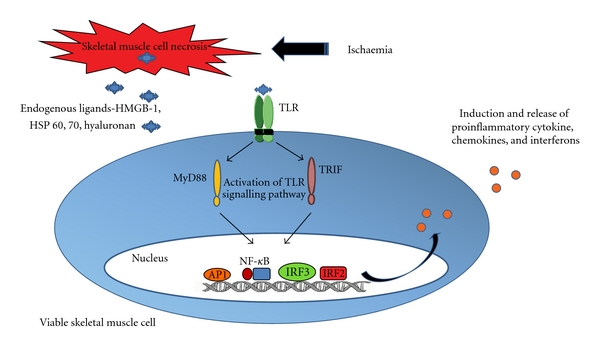
Proposed pathophysiological mechanism of skeletal muscle damage in CLI. Skeletal muscle ischaemia initiates muscle cell apoptosis and necrosis leading to the release of endogenous ligands such as HMGB-1. Subsequently TLRs are activated in other viable muscle cells causing signalling through one or more TLR signalling pathways. This may lead to the activation of transcription factors such as NF-*κ*B, AP-1, IRF 3, and 2. The consequent activation of transcription factors leads to the induction and release of proinflammatory cytokines, chemokines, and interferons that propagate the skeletal muscle damage.

There is growing evidence to suggest that some of these destructive processes are involved in the pathophysiology of skeletal muscle damage in CLI, and TLRs are implicated in mediating this damage. However, further studies are required to elaborate on the preliminary data including the identification of the key TLRs involved in mediating skeletal muscle damage in CLI (Figure 3). One can speculate that TLR 2 and TLR 4 may be important as there is extensive data on the role of these two receptors in mediating ischaemic and I/R injury. However, other TLRs such as TLR 1 and 6 are promising targets as they are known to heterodimerise with TLR 2 [16]. It would also be essential to identify the particular TLR signalling pathways and transcription factors involved in ischaemic skeletal muscle. Further TLR antagonists are under clinical development in the treatment of a number of inflammatory and autoimmune diseases [19], and TLR antagonists have also been shown to reduce ischaemia-induced injury. A better understanding of the pathophysiology of CLI with concomitant development of TLR antagonists may identify treatment modalities that can be translated into clinical benefit for patients with CLI.

## Figures and Tables

**Table 1:** Exogenous/endogenous ligands and antagonists of TLRs; n.d.: not discovered.

TLR	Microbial ligands	Endogenous ligands	Antagonists
TLR1/TLR2	Triacyl lipopeptides (Pam3CSK4)	n.d.	n.d.
TLR2/TLR6	Diacyl lipopeptides (Pam2CSK4), zymosan, porins, bacterial peptidoglycan, LPSs of gram positive bacteria	HSP-60, HSP-70, HMGB-1	n.d.
TLR3	Ds RNA	mRNA	n.d.
TLR4	LPS	HSP-22, HSP-60, HSP-70, HSP-96, fibrinogen, HMGB-1, hyaluronan fragments, fibronectin (extra domain A)	Eritoran (E5564, a lipid A derivative), TAK-242
TLR5	Flagellin	n.d.	n.d.
TLR7	ssRNA (viral)	ssRNA (immune complexes)	CpG ODN, CpG 52364
TLR8	ssRNA (viral)	ssRNA (immune complexes)	CpG 52364
TLR9	DNA (bacterial/viral)	DNA (immune complexes)	CpG ODN, CpG 52364
TLR11	Toxoplasma gondii	n.d.	n.d.
TLR10, 12, 13	n.d.	n.d.	n.d.

**Table 2:** Summary of the evidence for the role of TLRs in the pathogenesis of tissue damage in ischaemia and I/R injury.

Organ ischaemia and I/R injury	Expression of TLRs	Upregulation of TLRs in ischaemia	Evidence for role of TLR in pathophysiology	Potential endogenous ligand implicated in pathogenesis
Cerebral	Glial cells: TLR 1–9 [32, 33] Neurons: TLR 2 and 4 [34]	TLR 2, 4 and 9 [34, 35]	TLR 2 and 4 knockout mice have reduced infarct size following ischaemia [34, 36–38]	HSP 70 [34]

Liver	Hepatocytes: TLR 2, 3, 4 and 5 [39] Non-parenchymal cells: TLR 2, 3, 4, 5, 7 and 9 [39]	TLR 2 [40]	TLR 4 and 9 knockout mice are protected against ischaemia-induced liver injury [41–43]	HSP 72 [44] and HMGB-1 [42, 45]

Renal	Parenchyma: TLRs 1–10 at varying detection levels [46–48]	TLR 2 and 4 [47, 49]	TLR 2 and 4 knockout mice are protected against renal I/R injury, and this is associated with a reduction in inflammatory cytokine levels [49–52]	HMGB-1, hyaluronan and biglycan [49, 51, 53]

Myocardial	Myocytes: TLR 2, 3, 4 and 6 [54]	TLR 4 [55]	TLR 2- and 4-deficient mice show reduced myocardial infarct size [56–59]. TLR 4 antagonist eritoran leads to reduced infarct size, NF-*κ*B nuclear translocation, and proinflammatory cytokine expression [60]	HMGB-1 [61]

Skeletal muscle	TLR 1–9 [62–64]	TLR 2, 4, 6 [65]	TLR 2 antagonsim reduces pro-inflammatory cytokine expression in an *in vitro *model of skeletal muscle ischaemia [65]	Under investigation

## References

[1] Varu VN, Hogg ME, Kibbe MR (2010). Critical limb ischemia. *Journal of Vascular Surgery*.

[2] Bertelè V, Roncaglioni MC, Pangrazzi J, Terzian E (1999). Clinical outcome and its predictors in 1560 patients with critical leg ischaemia. *European Journal of Vascular and Endovascular Surgery*.

[3] Cieri E, Lenti M, De Rango P, Isernia G, Marucchini A, Cao P (2011). Functional ability in patients with critical limb ischaemia is unaffected by successful revascularisation. *European Journal of Vascular and Endovascular Surgery*.

[4] Nicoloff AD, Taylor L.M. Jr, McLafferty RB, Moneta GL, Porter JM, Menzoian JO (1998). Patient recovery after infrainguinal bypass grafting for limb salvage. *Journal of Vascular Surgery*.

[5] Dormandy JA (2000). Two randomised and placebo-controlled studies of an oral prostacyclin analogue (Iloprost) in severe leg ischaemia. *European Journal of Vascular and Endovascular Surgery*.

[6] Arumugam TV, Okun E, Tang SC, Thundyil J, Taylor SM, Woodruff TM (2009). Toll-like receptors in ischemia-reperfusion injury. *Shock*.

[7] Akira S, Takeda K (2004). Toll-like receptor signalling. *Nature Reviews Immunology*.

[8] Frangogiannis NG (2007). Chemokines in ischemia and reperfusion. *Thrombosis and Haemostasis*.

[9] Takeda K, Kaisho T, Akira S (2003). Toll-like receptors. *Annual Review of Immunology*.

[10] Hadley JS, Wang JE, Michaels LC (2007). Alterations in inflammatory capacity and TLR expression on monocytes and neutrophils after cardiopulmonary bypass. *Shock*.

[11] Gerondakis S, Grumont RJ, Banerjee A (2007). Regulating B-cell activation and survival in response to TLR signals. *Immunology and Cell Biology*.

[12] Breslin JW, Wu MH, Guo M, Reynoso R, Yuan SY (2008). Toll-like receptor 4 contributes to microvascular inflammation and barrier dysfunction in thermal injury. *Shock*.

[13] Boyd JH, Mathur S, Wang Y, Bateman RM, Walley KR (2006). Toll-like receptor stimulation in cardiomyoctes decreases contractility and initiates an NF-*κ*B dependent inflammatory response. *Cardiovascular Research*.

[14] Frost RA, Nystrom GJ, Lang CH (2006). Multiple Toll-like receptor ligands induce an IL-6 transcriptional response in skeletal myocytes. *American Journal of Physiology—Regulatory Integrative and Comparative Physiology*.

[15] Jong SP, Gamboni-Robertson F, He Q (2006). High mobility group box 1 protein interacts with multiple Toll-like receptors. *American Journal of Physiology—Cell Physiology*.

[16] Nakao Y, Funami K, Kikkawa S (2005). Surface-expressed TLR6 participates in the recognition of diacylated lipopeptide and peptidoglycan in human cells. *Journal of Immunology*.

[17] Tsan MF, Gao B (2004). Endogenous ligands of Toll-like receptors. *Journal of Leukocyte Biology*.

[18] Termeer C, Benedix F, Sleeman J (2002). Oligosaccharides of hyaluronan activate dendritic cells via Toll-like receptor 4. *Journal of Experimental Medicine*.

[19] Kanzler H, Barrat FJ, Hessel EM, Coffman RL (2007). Therapeutic targeting of innate immunity with Toll-like receptor agonists and antagonists. *Nature Medicine*.

[20] Akira S, Takeda K (2004). Toll-like receptor signalling. *Nature Reviews Immunology*.

[21] Karin M, Ben-Neriah Y (2000). Phosphorylation meets ubiquitination: the control of NF-*κ*B activity. *Annual Review of Immunology*.

[22] Kawai T, Akira S (2006). TLR signaling. *Cell Death and Differentiation*.

[23] Fitzgerald KA, McWhirter SM, Faia KL (2003). IKKE and TBKI are essential components of the IRF3 signalling pathway. *Nature Immunology*.

[24] Kawai T, Akira S (2007). TLR signaling. *Seminars in Immunology*.

[25] Jordan JE, Zhao ZQ, Vinten-Johansen J (1999). The role of neutrophils in myocardial ischemia-reperfusion injury. *Cardiovascular Research*.

[26] Yilmaz G, Arumugam TV, Stokes KY, Granger DN (2006). Role of T lymphocytes and interferon-*γ* in ischemic stroke. *Circulation*.

[27] Thurman JM (2007). Triggers of inflammation after renal ischemia/reperfusion. *Clinical Immunology*.

[28] Boros P, Bromberg JS (2006). New cellular and molecular immune pathways in ischemia/reperfusion injury. *American Journal of Transplantation*.

[29] Collard CD, Vakeva A, Morrissey MA (2000). Complement activation after oxidative stress: role of the lectin complement pathway. *American Journal of Pathology*.

[30] Broughton BRS, Reutens DC, Sobey CG (2009). Apoptotic mechanisms after cerebral ischemia. *Stroke*.

[31] Aliprantis AO, Yang RB, Weiss DS, Godowski P, Zychlinsky A (2000). The apoptotic signaling pathway activated by Toll-like receptor-2. *EMBO Journal*.

[32] Bsibsi M, Ravid R, Gveric D, Van Noort JM (2002). Broad expression of Toll-like receptors in the human central nervous system. *Journal of Neuropathology and Experimental Neurology*.

[33] Jack CS, Arbour N, Manusow J (2005). TLR signaling tailors innate immune responses in human microglia and astrocytes. *Journal of Immunology*.

[34] Tang SC, Arumugam TV, Xu X (2007). Pivotal role for neuronal Toll-like receptors in ischemic brain injury and functional deficits. *Proceedings of the National Academy of Sciences of the United States of America*.

[35] Stevens SL, Ciesielski TMP, Marsh BJ (2008). Toll-like receptor 9: a new target of ischemic preconditioning in the brain. *Journal of Cerebral Blood Flow and Metabolism*.

[36] Kilic U, Kilic E, Matter CM, Bassetti CL, Hermann DM (2008). TLR-4 deficiency protects against focal cerebral ischemia and axotomy-induced neurodegeneration. *Neurobiology of Disease*.

[37] Lehnardt S, Lehmann S, Kaul D (2007). Toll-like receptor 2 mediates CNS injury in focal cerebral ischemia. *Journal of Neuroimmunology*.

[38] Cao CX, Yang QW, Lv FL, Cui J, Fu HB, Wang JZ (2007). Reduced cerebral ischemia-reperfusion injury in Toll-like receptor 4 deficient mice. *Biochemical and Biophysical Research Communications*.

[39] Seki E, Brenner DA (2008). Toll-like receptors and adaptor molecules in liver disease: update. *Hepatology*.

[40] Zhang J, Wu H, Wang L, Zhang J, Wang H, Zheng Q (2004). TLR2 mRNA upregulation in ischemic lobes in mouse partial hepatic ischemia/reperfusion injury model. *Journal of Huazhong University of Science and Technology—Medical Science*.

[41] Tsung A, Hoffman RA, Izuishi K (2005). Hepatic ischemia/reperfusion injury involves functional TLR4 signaling in nonparenchymal cells. *Journal of Immunology*.

[42] Bamboat ZM, Balachandran VP, Ocuin LM, Obaid H, Plitas G, DeMatteo RP (2010). Toll-like receptor 9 inhibition confers protection from liver ischemia-reperfusion injury. *Hepatology*.

[43] Shen X-D, Ke B, Zhai Y (2005). Toll-like receptor and heme oxygenase-1 signaling in hepatic ischemia/reperfusion injury. *American Journal of Transplantation*.

[44] Okaya T, Blanchard J, Schuster R (2005). Age-dependent responses to hepatic ischemia/reperfusion injury. *Shock*.

[45] Tsung A, Sahai R, Tanaka H (2005). The nuclear factor HMGB1 mediates hepatic injury after murine liver ischemia-reperfusion. *Journal of Experimental Medicine*.

[46] Patole PS, Pawar RD, Lech M (2006). Expression and regulation of Toll-like receptors in lupus-like immune complex glomerulonephritis of MRL-Fas(lpr) mice. *Nephrology Dialysis Transplantation*.

[47] Wolfs TGAM, Buurman WA, Van Schadewijk A (2002). In vivo expression of Toll-like receptor 2 and 4 by renal epithelial cells: IFN-*γ* and TNF-*α* mediated up-regulation during inflammation. *Journal of Immunology*.

[48] Schröppel B, He JC (2006). Expression of Toll-like receptors in the kidney: their potential role beyond infection. *Kidney International*.

[49] Wu H, Chen G, Wyburn KR (2007). TLR4 activation mediates kidney ischemia/reperfusion injury. *Journal of Clinical Investigation*.

[50] Rusai K, Sollinger D, Baumann M (2010). Toll-like receptors 2 and 4 in renal ischemia/reperfusion injury. *Pediatric Nephrology*.

[51] Leemans JC, Stokman G, Claessen N (2005). Renal-associated TLR2 mediates ischemia/reperfusion injury in the kidney. *Journal of Clinical Investigation*.

[52] Pulskens WP, Teske GJ, Butter LM (2008). Toll-like receptor-4 coordinates the innate immune response of the kidney to renal ischemia/reperfusion injury. *PLoS ONE*.

[53] Krüger B, Krick S, Dhillon N (2009). Donor toll-like receptor 4 contributes to ischemia and reperfusion injury following human kidney transplantation. *Proceedings of the National Academy of Sciences of the United States of America*.

[54] Woo YJ, Taylor MD, Cohen JE (2004). Ethyl pyruvate preserves cardiac function and attenuates oxidative injury after prolonged myocardial ischemia. *Journal of Thoracic and Cardiovascular Surgery*.

[55] de Kleijn D, Pasterkamp G (2003). Toll-like receptors in cardiovascular diseases. *Cardiovascular Research*.

[56] Frantz S, Kobzik L, Kim YD (1999). Toll4 (TLR4) expression in cardiac myocytes in normal and failing myocardium. *Journal of Clinical Investigation*.

[57] Chong AJ, Shimamoto A, Hampton CR (2004). Toll-like receptor 4 mediates ischemia/reperfusion injury of the heart. *Journal of Thoracic and Cardiovascular Surgery*.

[58] Kim SC, Ghanem A, Stapel H (2007). Toll-like receptor 4 deficiency: smaller infarcts, but nogain in function. *BMC Physiology*.

[59] Shimamoto A, Chong AJ, Yada M (2006). Inhibition of toll-like receptor 4 with eritoran attenuates myocardial ischemia-reperfusion injury. *Circulation*.

[60] Frantz S, Tillmanns J, Kuhlencordt PJ (2007). Tissue-specific effects of the nuclear factor *κ*B subunit p50 on myocardial ischemia-reperfusion injury. *American Journal of Pathology*.

[61] Warren GL, Hulderman T, Liston A, Simeonova PP (2011). Toll-like and adenosine receptor expression in injured skeletal muscle. *Muscle and Nerve*.

[62] Visser M, Pahor M, Taaffe DR (2002). Relationship of interleukin-6 and tumor necrosis factor-*α* with muscle mass and muscle strength in elderly men and women: the health ABC study. *Journals of Gerontology—Series A Biological Sciences and Medical Sciences*.

[63] Meadows KA, Holly JMP, Stewart CEH (2000). Tumor necrosis factor-*α*-induced apoptosis is associated with suppression of insulin-like growth factor binding protein-5 secretion in differentiating murine skeletal myoblasts. *Journal of Cellular Physiology*.

[64] Frost RA, Nystrom GJ, Lang CH (2006). Multiple Toll-like receptor ligands induce an IL-6 transcriptional response in skeletal myocytes. *American Journal of Physiology—Regulatory Integrative and Comparative Physiology*.

[65] Patel H, Shiwen X (2011). Toll-like receptor 2 antagonism reduces inflammatory cytokine release in skeletal muscle in vitro. *British Journal of Surgery*.

[66] van Noort JM, Bsibsi M (2009). Toll-like receptors in the CNS: implications for neurodegeneration and repair. *Progress in Brain Research*.

[67] Caso JR, Pradillo JM, Hurtado O, Lorenzo P, Moro MA, Lizasoain I (2007). Toll-like receptor 4 is involved in brain damage and inflammation after experimental stroke. *Circulation*.

[68] Shibayama Y, Asaka S, Nishijima A (1991). Mechanism of liver injury following ischemia. *Experimental and Molecular Pathology*.

[69] Montalvo-Jave EE, Escalante-Tattersfield T, Ortega-Salgado JA, Piña E, Geller DA (2008). Factors in the pathophysiology of the liver ischemia-reperfusion injury. *Journal of Surgical Research*.

[70] Tsukamoto H (2002). Redox regulation of cytokine expression in Kupffer cells. *Antioxidants and Redox Signaling*.

[71] Jaeschke H, Farhood A, Bautista AP, Spolarics Z, Spitzer JJ (1993). Complement activates Kupffer cells and neutrophils during reperfusion after hepatic ischemia. *American Journal of Physiology—Gastrointestinal and Liver Physiology*.

[72] Anaya-Prado R, Toledo-Pereyra LH, Lentsch AB, Ward PA (2002). Ischemia/reperfusion injury. *Journal of Surgical Research*.

[73] Li Y, Zhang W, Mantell LL, Kazzaz JA, Fein AM, Horowitz S (1997). Nuclear factor-*κ*B is activated by hyperoxia but does not protect from cell death. *Journal of Biological Chemistry*.

[74] Wullaert A, van Loo G, Heyninck K, Beyaert R (2007). Hepatic tumor necrosis factor signaling and nuclear factor-*κ*B: effects on liver homeostasis and beyond. *Endocrine Reviews*.

[75] Rüdiger HA, Clavien P (2002). Tumor necrosis factor *α*, but not Fas, mediates hepatocellular apoptosis in the murine ischemic liver. *Gastroenterology*.

[76] Gujral JS, Bucci TJ, Farhood A, Jaeschke H (2001). Mechanism of cell death during warm hepatic ischemia-reperfusion in rats: apoptosis or necrosis?. *Hepatology*.

[77] Arumugam TV, Shiels IA, Woodruff TM, Granger DN, Taylor SM (2004). The role of the complement system in ischemia-reperfusion injury. *Shock*.

[78] Galloway E, Shin T, Huber N (2008). Activation of hepatocytes by extracellular heat shock protein 72. *American Journal of Physiology—Cell Physiology*.

[79] Lentsch AB, Yoshidome H, Cheadle WG, Miller FN, Edwards MJ (1998). Chemokine involvement in hepatic ischemia/reperfusion injury in mice: roles for macrophage inflammatory protein-2 and KC. *Hepatology*.

[80] Zhai Y, Shen XD, O’Connell R (2004). Cutting edge: TLR4 activation mediates liver ischemia/reperfusion inflammatory response via IFN regulatory factor 3-dependent MyD88-independent pathway. *Journal of Immunology*.

[81] Lieberthal W, Menza SA, Levine JS (1998). Graded ATP depletion can cause necrosis or apoptosis of cultured mouse proximal tubular cells. *American Journal of Physiology—Renal Physiology*.

[82] Takada M, Nadeau KC, Shaw GD, Marquette KA, Tilney NL (1997). The cytokine-adhesion molecule cascade in ischemia/reperfusion injury of the rat kidney. Inhibition by a soluble P-selectin ligand. *Journal of Clinical Investigation*.

[83] Thurman JM, Ljubanovic D, Edelstein CL, Gilkeson GS, Holers VM (2003). Lack of a functional alternative complement pathway ameliorates ischemic acute renal failure in mice. *Journal of Immunology*.

[84] Singbartl K, Green SA, Klaus L (2000). Blocking P-selectin protects from ischemia/reperfusion-induced acute renal failure. *FASEB Journal*.

[85] Burne-Taney MJ, Ascon DB, Daniels F, Racusen L, Baldwin W, Rabb H (2003). B cell deficiency confers protection from renal ischemia reperfusion injury. *Journal of Immunology*.

[86] Ysebaert DK, De Greef KE, De Beuf A (2004). T cells as mediators in renal ischemia/reperfusion injury. *Kidney International*.

[87] Shigeoka AA, Holscher TD, King AJ (2007). TLR2 is constitutively expressed within the kidney and participates in ischemic renal injury through both myD88-dependent and -independent pathways. *Journal of Immunology*.

[88] Maxwell SRJ, Lip GYH (1997). Reperfusion injury: a review of the pathophysiology, clinical manifestations and therapeutic options. *International Journal of Cardiology*.

[89] Buja LM (2005). Myocardial ischemia and reperfusion injury. *Cardiovascular Pathology*.

[90] Otani H (2008). Ischemic preconditioning: from molecular mechanisms to therapeutic opportunities. *Antioxidants and Redox Signaling*.

[91] Kin H, Wang NP, Mykytenko J (2008). Inhibition of myocardial apoptosis by postconditioning is associated with attenuation of oxidative stress-mediated nuclear factor-*κ*B translocation and TNF*α* release. *Shock*.

[92] Ikonomidis I, Andreotti F, Economou E, Stefanadis C, Toutouzas P, Nihoyannopoulos P (1999). Increased proinflammatory cytokines in patients with chronic stable angina and their reduction by aspirin. *Circulation*.

[93] Hennein HA, Ebba H, Rodriguez JL (1994). Relationship of the proinflammatory cytokines to myocardial ischemia and dysfunction after uncomplicated coronary revascularization. *Journal of Thoracic and Cardiovascular Surgery*.

[94] Frantz S, Kelly RA, Bourcier T (2001). Role of TLR-2 in the activation of nuclear factor *κ*B by oxidative stress in cardiac myocytes. *Journal of Biological Chemistry*.

[95] Oyama JI, Blais C, Liu X (2004). Reduced myocardial ischemia-reperfusion injury in Toll-Like receptor 4-deficient mice. *Circulation*.

[96] Hua F, Ha T, Ma J (2007). Protection against myocardial ischemia/reperfusion injury in TLR4-deficient mice is mediated through a phosphoinositide 3-kinase-dependent mechanism. *Journal of Immunology*.

[97] Shishido T, Nozaki N, Yamaguchi S (2003). Toll-Like receptor-2 modulates ventricular remodeling after myocardial infarction. *Circulation*.

[98] Favre J, Musette P, Douin-Echinard V (2007). Toll-like receptors 2-deficient mice are protected against postischemic coronary endothelial dysfunction. *Arteriosclerosis, Thrombosis, and Vascular Biology*.

[99] Andrassy M, Volz HC, Igwe JC (2008). High-mobility group box-1 in ischemia-reperfusion injury of the heart. *Circulation*.

[100] Cheng TO (2006). All teas are not created equal: the Chinese green tea and cardiovascular health. *International Journal of Cardiology*.

[101] Wang P (2001). Andrenomedullin and cardiovascular responses in sepsis. *Peptides*.

[102] Belkin M, Brown RD, Wright JG, LaMorte WW, Hobson RW (1988). A new quantitative spectrophotometric assay of ischemia-reperfusion injury in skeletal muscle. *American Journal of Surgery*.

[103] Pipinos II, Judge AR, Selsby JT (2008). The myopathy of peripheral arterial occlusive disease: part 2. Oxidative stress, neuropathy, and shift in muscle fiber type. *Vascular and Endovascular Surgery*.

[104] Hayes G, Liauw S, Romaschin A, Walker PM (1988). Separation of reperfusion injury from ischemia-induced necrosis. *Surgical Forum*.

[105] Mitchell RG, Duscha BD, Robbins JL (2007). Increased levels of apoptosis in gastrocnemius skeletal muscle in patients with peripheral arterial disease. *Vascular Medicine*.

[106] Signorelli SS, Mazzarino MC, Di Pino L (2003). High circulating levels of cytokines (IL-6 and TNF*α*), adhesion molecules (VCAM-1 and ICAM-1) and selectins in patients with peripheral arterial disease at rest and after a treadmill test. *Vascular Medicine*.

[107] Goodman MN (1991). Tumor necrosis factor induces skeletal muscle protein breakdown in rats. *American Journal of Physiology—Endocrinology and Metabolism*.

[108] Goodman MN (1994). Interleukin-6 induces skeletal muscle protein breakdown in rats. *Proceedings of the Society for Experimental Biology and Medicine*.

[109] Lang CH, Silvis C, Deshpande N, Nystrom G, Frost RA (2003). Endotoxin stimulates in vivo expression of inflammatory cytokines tumor necrosis factor alpha, interleukin-1beta, -6, and high-mobility-group protein-1 in skeletal muscle. *Shock*.

[110] Grundtman C, Bruton J, Yamada T (2010). Effects of HMGB1 on in vitro responses of isolated muscle fibers and functional aspects in skeletal muscles of idiopathic inflammatory myopathies. *FASEB Journal*.

[111] Liu Y, Steinacker JM (2001). Changes in skeletal muscle heat shock proteins: pathological significance. *Frontiers in Bioscience*.

